# Predicting long-term outcome of Internet-delivered cognitive behavior therapy for social anxiety disorder using fMRI and support vector machine learning

**DOI:** 10.1038/tp.2015.22

**Published:** 2015-03-17

**Authors:** K N T Månsson, A Frick, C-J Boraxbekk, A F Marquand, S C R Williams, P Carlbring, G Andersson, T Furmark

**Affiliations:** 1 Division of Psychology, Department of Behavioural Sciences and Learning, Linköping University, Linköping, Sweden; 2 Department of Psychology, Uppsala University, Uppsala, Sweden; 3 Centre for Population Studies, Ageing and Living Conditions, Umeå University, Umeå, Sweden; 4 Umeå Center for Functional Brain Imaging (UFBI), Umeå University, Umeå, Sweden; 5 Donders Institute for Brain, Cognition and Behaviour, Radboud University, Nijmegen, The Netherlands; 6 Department of Neuroimaging, Centre for Neuroimaging Sciences, Institute of Psychiatry, King's College London, London, UK; 7 Department of Psychology, Stockholm University, Stockholm, Sweden; 8 Psychiatry Section, Department of Clinical Neuroscience, Karolinska Institutet, Stockholm, Sweden

## Abstract

Cognitive behavior therapy (CBT) is an effective treatment for social anxiety disorder (SAD), but many patients do not respond sufficiently and a substantial proportion relapse after treatment has ended. Predicting an individual's long-term clinical response therefore remains an important challenge. This study aimed at assessing neural predictors of long-term treatment outcome in participants with SAD 1 year after completion of Internet-delivered CBT (iCBT). Twenty-six participants diagnosed with SAD underwent iCBT including attention bias modification for a total of 13 weeks. Support vector machines (SVMs), a supervised pattern recognition method allowing predictions at the individual level, were trained to separate long-term treatment responders from nonresponders based on blood oxygen level-dependent (BOLD) responses to self-referential criticism. The Clinical Global Impression-Improvement scale was the main instrument to determine treatment response at the 1-year follow-up. Results showed that the proportion of long-term responders was 52% (12/23). From multivariate BOLD responses in the dorsal anterior cingulate cortex (dACC) together with the amygdala, we were able to predict long-term response rate of iCBT with an accuracy of 92% (confidence interval 95% 73.2–97.6). This activation pattern was, however, not predictive of improvement in the continuous Liebowitz Social Anxiety Scale—Self-report version. Follow-up psychophysiological interaction analyses revealed that lower dACC–amygdala coupling was associated with better long-term treatment response. Thus, BOLD response patterns in the fear-expressing dACC–amygdala regions were highly predictive of long-term treatment outcome of iCBT, and the initial coupling between these regions differentiated long-term responders from nonresponders. The SVM-neuroimaging approach could be of particular clinical value as it allows for accurate prediction of treatment outcome at the level of the individual.

## Introduction

Social anxiety disorder (SAD) is a common^1^ and disabling disorder that often precedes other serious mental health problems such as depression.^2^ SAD is associated with aberrant information-processing and cognitive biases toward negative information regarding the self, for example, self-focused attention.^3^ Cognitive behavior therapy (CBT), including modification of cognitive biases, is an effective treatment for SAD,^4^ and can be delivered in accessible formats, for example, via the Internet.^5^ Internet-delivered CBT (iCBT) has been evaluated in several randomized controlled studies at different sites,^5^ and sustained effects have been observed up to 5 years later.^6^ Also, CBT for SAD has been shown to be equally effective when delivered via the Internet in comparison with face-to-face group treatment.^7^ However, significant proportions of the treated patients relapse over time or do not respond sufficiently.^8^ Accordingly, improving long-term treatment outcome of CBT remains an important challenge, and factors that reliably predict lasting therapeutic success need to be identified.

Recent functional magnetic resonance imaging (fMRI) studies suggest that neural biomarkers add substantial value to predictions of CBT outcome. In SAD participants, Doehrmann *et al.*^9^ demonstrated that initial activations of the visual cortex, in response to emotional face stimuli, predicted symptom improvement with CBT, and that brain measures vastly improved prediction success in comparison with other clinical variables. Klumpp *et al.*,^10^ using a similar task, found that pretreatment reactivity in the inferior frontal and the superior and middle temporal gyri was associated with reduced social anxiety following CBT. However, both studies evaluated the short-term treatment response only. In a previous positron emission tomography study from our lab, the initial attenuation (pre–post) of anxiety-related amygdala activity in treated SAD participants was associated with clinical improvement 1 year later, but as the sample was small the study could not discriminate properly between the effects of CBT and selective serotonin reuptake inhibitors (SSRIs).^11^

Mass-univariate voxel-wise methods, seeking pretreatment brain voxels that correlate with symptom improvement or differ between responders and nonresponders, have so far been the most common approach in psychiatric prediction studies. However, such studies may not be easily translated to clinical settings when the concern is whether or not a certain patient will respond to a specific treatment. In contrast, supervised pattern recognition methods constitute a novel approach in clinical neuroimaging, utilizing patterns of information across many voxels, for example, to separate responders from nonresponders.^12^ In pattern recognition, a statistical model is estimated on a subset of the data (‘training') and then applied to predict the diagnostic or prognostic label of novel unseen data (‘testing'). This is usually achieved within a cross-validation procedure, whereby the model is repeatedly retrained, withholding a different partition of data each time. This approach is standard within the statistical literature and is well known to provide approximately unbiased estimates of generalizability to new samples, and helps to minimize the possibility of ‘over-fitting' the data. Of crucial importance, and in contrast to voxel-wise group statistics, pattern recognition analyses can make predictions at the level of the individual based on the pattern within the data, for example, treatment responder status or diagnostic category for a new unseen participant.^12, 13^

A supervised pattern recognition method called support vector machine (SVM)^13^ was recently successful in separating SAD from panic disorder participants, and from healthy controls.^14^ In addition, we recently demonstrated the utility of SVM and accurately discriminated SAD participants from healthy controls based on the multivariate pattern of blood oxygen level-dependent (BOLD) response in the fear network,^15^ that is, the amygdala, hippocampus, anterior cingulate cortex (ACC) and insula, frequently demonstrated to be dysfunctional in SAD.^16^ Pretreatment reactivity in fear network regions has also been associated with subsequent treatment outcome in SAD and other anxiety disorders.^17, 18^ The ACC in particular has been implicated in treatment prediction studies of depression,^19^ and anxiety.^20^ Moreover, cognitive control of emotion, involving prefrontal–limbic interactions,^21^ has been suggested to be impaired in participants with SAD and restored with CBT^22^ but it is not known whether such interactions are predictive of treatment response in SAD. Although the SVM approach has been successful in predicting treatment response in depression,^23^ there are, to our knowledge, no studies using SVM to predict CBT treatment outcome in anxiety disorders such as SAD. Also, neuroimaging prediction studies of long-term treatment effects are largely lacking in psychiatric research.^24^

The objective of the present study was to use SVM classification to evaluate neural predictors of long-term iCBT response 1 year after treatment of participants with SAD. In addition to iCBT, the participants underwent Internet-delivered attention bias modification (ABM) in a cross-over design.^25^ Adding ABM has not been shown to further improve outcome,^26^ and we refer to the present combined intervention as iCBT. Using a disorder-relevant fMRI paradigm, we entered BOLD responses to sentences with negative content about oneself, that is, self-referential criticism^27^ into linear SVMs to classify responder status at long-term follow-up. Our SVM analyses focused primarily on the fear network and brain regions associated with cognitive control of negative emotion, that is, the ACC, amygdala, hippocampus, insula, ventromedial prefrontal cortex and dorsolateral prefrontal cortex.

## Materials and methods

### Participants

This study included 26 right-handed participants with a primary diagnosis of SAD (85% having the generalized subtype, recruited via media advertisements (see Table 1 and Supplementary Figure S1 in Supplementary Material). The sample, including inclusion/exclusion criteria, has been described in detail elsewhere,^25^ and the screening procedure was similar to our previous treatment studies.^5, 28^ Briefly, participants reported interest on a webpage and answered self-report questionnaires via the Internet regarding social anxiety, depression and magnetic resonance safety. Participants had no neurological or major somatic disorder, no suicidal ideation, no other ongoing psychological treatment and they were not included if psychotropic medication (for example, SSRIs) was recently initiated or changed, that is, a stable dose for 3 months was required. Applicants fulfilling the initial screening criteria were interviewed via telephone using the structured clinical interview for the 4th version of Diagnostic and Statistical Manual of Mental Disorders (DSM-IV) axis 1 (SCID-I).^29^ At baseline, 8 (31%, 8/26) participants were currently on prescription medication, that is, SSRIs. Five participants had a history of SSRI treatment and 13 were medication-naive. One participant (deemed as nonresponder) increased the dose from 50–100 mg sertraline from posttreatment to 1-year follow-up. Three participants withdrew and did not take part in the 1-year follow-up.

All participants gave written informed consent prior to participation. The study was conducted in accordance with the Declaration of Helsinki and approval was obtained from the regional ethic committee. The study was registered at ClinicalTrials.gov (ID: NCT01312571).

### Assessment of clinical response

Treatment response at 1-year follow-up was assessed with the Clinical Global Impression-Improvement (CGI-I) interview scale.^30^ In accordance with prior studies,^30^ scores of 1 or 2 (very much or much improved) defined treatment responders, whereas participants scoring ⩾3 (ranging from minimally improved to very much worse) were classified as nonresponders. Two psychologists, blind to the experimental conditions, conducted the interviews. In addition, we predicted long-term clinical improvement (pre follow-up) using the continuous Liebowitz Social Anxiety Scale—Self-report version, LSAS-SR.^31^

### Treatment

This study included two forms of intervention for SAD. Both treatments were provided to all participants in a cross-over design where half of the participants started with iCBT and the other half started with ABM.^25^ The guided iCBT protocol contained a 9-week intervention supported by a clinician and has been found effective in several randomized controlled trials.^5, 28, 32, 33^ Previously, we showed that the short-term outcome in this sample was in favor of iCBT, that is, 66% of the iCBT participants, in comparison with 25% of the ABM group, were deemed as CGI-I responders.^25^ At the 1-year follow-up, we found no effect of the order (*χ*^2^=0.4, *P*=0.51) in which the two interventions were presented.

Briefly, the treatment program is based upon the cognitive model of SAD described by Clark and Wells.^3^ The self-help text included information, exercises, homework assignments and ended with essay questions that were sent to the therapist. For more details, see Andersson *et al.*^5^ and Supplementary Material. The second intervention was ABM delivered over 4 weeks via the Internet, with exercises implemented twice a week, totaling eight sessions. ABM aims at changing the distorted attentional process characterizing anxiety disorders, that is, hypervigilance, or problem of disengagement from fearful stimuli.^34^ The Internet-delivered ABM used in this study was a web-based flash program displayed in full-screen mode at the participant's computer. E-mail and mobile phone SMS reminders prompted for each training session, scheduled on Tuesdays and Thursdays over the 4-week treatment course. The participant and therapist had no other communication during the treatment period.

### Experimental task

We evaluated BOLD responses to self-referential criticism using a disorder-relevant paradigm developed by Blair *et al.*,^27^ which was translated into Swedish. The sentences were split into three categories based on the referential target: self, other female or other male. The task included 216 sentences divided into three categories based on valence: negative (for example, ‘Nobody likes you'), neutral (for example, ‘You read') or positive (for example, ‘Everyone loves you') valences. Across valences, sentences were matched on number of letters and words, and appeared in randomized order. Participants were instructed to read the sentences and press a button with the right hand when they had read each sentence. Each sentence was presented for a maximum of 2500 ms. In addition, 96 fixation crosses (‘+') were randomly interspersed between the sentences and displayed for 2500 ms. Each sentence and fixation cross was separated by a cross or circle presented for 500 ms. Stimuli were demonstrated using the E-prime 2.0 software (Psychology Software Tools, Pittsburgh, PA, USA), projected on a screen and viewed through a tilted mirror attached to the head coil. The total duration of the task was 17 min and 20 s.

### Imaging

#### Structural and functional image acquisition

Structural high-resolution T1-weighted images (180 slices, 1 mm thickness, field of view: 250 mm, voxel size: 0.5 × 0.5 × 1 mm^3^) were collected prior to the functional images. BOLD contrast images (T2* weighted) were acquired using a 3T Discovery MR750 (General Electric, Madison, WI, USA) scanner equipped with a 32-channel head coil. The following scanning parameters were used: echo time: 30 ms, repetition time: 2000 ms, flip-angle: 80°, field of view: 25 × 25 cm^2^, matrix size: 96 × 96. Thirty-seven slices with a thickness of 3.4 mm (2.6 × 2.6 × 3.4 voxel size) were acquired every 2000 ms. Ten dummy scans were run before the image acquisition started to avoid signals resulting from progressive saturation. Preprocessing was done using Statistical Parametric Mapping Software 8 (SPM8; Wellcome Department of Cognitive Neurology, London, UK) implemented in MATLAB (Mathworks, Natick, MA, USA). Functional scans were realigned to the mean image of each run to correct for head movements during image acquisition, after which slice timing correction was performed. The functional scans (3 × 3 × 3 mm^3^ voxel dimensions) were then co-registered to the structural scans. Structural scans were segmented into gray and white matter, and parameters for warping the scans to the Montreal Neurological Institute (MNI152) template were calculated using the unified segmentation procedure in SPM8. Functional scans were subsequently warped to MNI standard space by applying these parameters, and smoothed with an 8-mm isotropic Gaussian kernel.

### Data analysis

Functional brain imaging data were fitted to the general linear model using SPM8. First-level, within-subject, analyses included nine regressors of interest in the model, valence (3) × target (3), containing onset times of respective sentences, as well as the isx movement parameters from the realignment preprocessing step as nuisance regressors. The model was convolved with the canonical hemodynamic response function defined in SPM8 and filtered using a 128-s high-pass filter. The other-referred contrast consisted of both female and male targets, and the contrast of interest used in subsequent analyses was self-referential criticism (self-negative minus other negative). On the basis of previous CBT prediction studies,^11, 17, 18^ we selected fear network regions of interest (ROIs) including the ACC, amygdala, hippocampus and insula. All regions were defined by the Automatic Anatomical Labeling ROI library within the Wake Forest University PickAtlas software.^35^ In follow-up analyses, the ACC was divided into the dorsal (dACC) and ventral (vACC) subdivisions by a split at the MNI-coordinate *z*=8, which corresponds to clusters 2 and 3 in Beckmann *et al.*^36^

We also investigated the predictive value of regions involved in cognitive control of negative emotion,^37^ including the dorsolateral prefrontal cortex (Brodmann areas 9 and 46) and ventromedial prefrontal cortex (10 mm radius spherical ROI centered at [*x*,*y*,*z*]=[4,32,−5]).^38^ Behavioral treatment effects and initial responder/nonresponder differences on demographic and clinical data were calculated using the STATA statistical software, v. 13.1 (Stata, College Station, TX, USA).

### Support vector machine learning

SVM analyses were carried out using the Pattern Recognition for Neuroimaging Toolbox.^39^ For each ROI or network, pretreatment contrast images of self-referential criticism were summarized using a dot-product kernel matrix, which was then used as input to a linear SVM. Each SVM was embedded within a leave-one-participant-out cross-validation framework and for each cross-validation fold, data were centered using the mean of the training data. The SVM soft margin parameter C was fixed to its default value 1.^39^ SVMs were trained to separate CGI-I responders from nonresponders at 1-year follow-up. We employed SVMs because they provide excellent performance for neuroimaging data relative to alternative approaches such as random forests.^40^ We tested the statistical significance of each classifier using permutation testing. This involved repeating the cross-validation procedure (1000 times) after randomly permuting the class labels. Significance of the classification accuracy was then derived by counting the number of times the permuted accuracy exceeded the true accuracy and dividing by 1000.

We used a discriminative mapping approach to visualize the relative contribution of the different brain regions to the classifier decision. This is a standard approach in SVM studies using neuroimaging data^39^ and was achieved by mapping the predictive weights in the original voxel space. It is important to emphasize that the voxel weights should not be interpreted as describing focal differences between classes in the same way as in mass-univariate analysis. Instead, the weights should be interpreted as a multivariate pattern, where the weight indicates the direction and quantifies the contribution of each voxel to the classifier decision. They do not permit inference at the level of individual voxels.

As a complement to SVM analyses on the dichotomous CGI-I measure, we also conducted relevance vector regressions^41^ in Pattern Recognition for Neuroimaging Toolbox with the aim to predict improvement (pre follow-up) on the continuous LSAS-SR.

In the main analyses we found the ACC to be highly predictive of long-term response. Because the ACC has strong anatomical connection with the amygdala,^42^ and because it is a functionally heterogeneous region, for example, with regard to control and expression of negative emotion,^43^ we analyzed the dACC and vACC subregions separately, and together with the amygdala. Furthermore, follow-up analyses (guided by the significant SVM classifications) using mass-univariate voxel-wise comparisons of initial BOLD response to self-referential criticism were performed between responders and nonresponders, and psychophysiological interactions^44^ were conducted using the dACC and vACC as seeds to evaluate couplings between these regions and the amygdala during self-referential criticism.

### Supplementary analyses

Additional SVM analyses were performed using an intention-to-treat approach, that is, all participants that withdrew from the study were deemed as nonresponders. We also excluded all participants with concurrent psychotropic medication. In addition, ROIs from two previously reported univariate prediction studies of short-term outcome of CBT for SAD^9, 10^ were evaluated, as well as whole-brain analysis. SVM prediction of SAD diagnostic status, assessed with the structured clinical interview for DSM-IV,^29^ was also performed. In addition, we checked for associations between descriptive variables (Table 1) and responder status, withdrawal and treatment compliance. Furthermore, the predictive value of descriptive (clinical and demographic) variables on long-term clinical response was assessed by multiple logistic regression analyses. Results of these analyses are reported in Supplementary Material.

## Results

### Prediction of long-term clinical response

There were 12/23 (52%) responders at 1-year follow-up according to the clinical interview CGI-I. Participants improved on the LSAS-SR from pretreatment to follow-up (*t*(23)=7.52, *P*<0.001, mean 75.37±19.1 to 44.67±22.8), and there was no significant change between the posttreatment and 1-year follow-up assessments (*t*(23)=0.70, *P=*0.489).^25^

**Figure 1:**
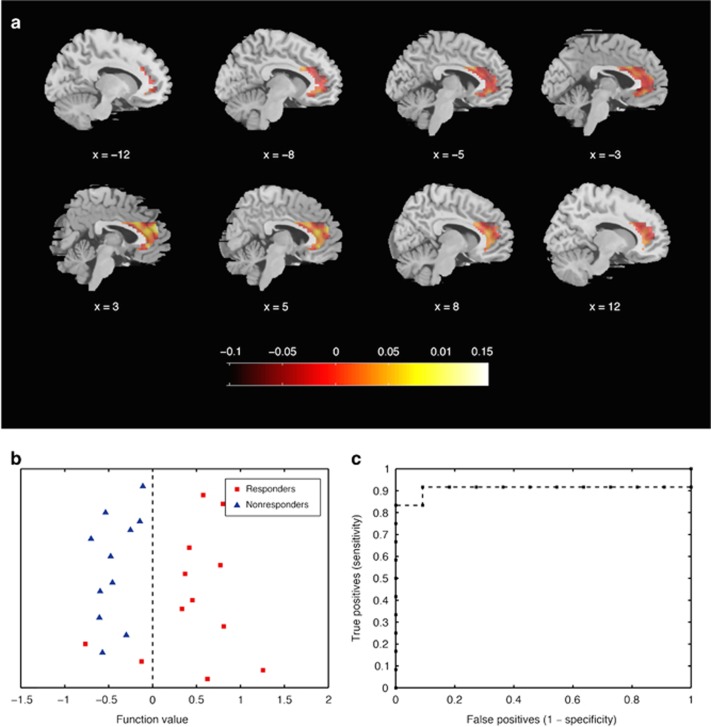
Support vector machine classification of responder status at 1-year follow-up in the anterior cingulate cortex. (**a**) Weight map indicating relative weights ascribed to voxels at representative sagittal slices. (**b**) Classification of responder status. (**c**) Receiver-operating characteristic curve, including area under the curve (AUC=0.91).

SVM analyses of pretreatment BOLD response to self-referential criticism (self>other referred criticism) showed that information from the ACC was highly accurate in classifying CGI-I responder status 1 year after treatment (balanced accuracy 91.7%, confidence interval 95% 73.2–97.6; Table 2 and Figure 1). Relevance vector regression predictions of improvement on the LSAS-SR were not significant (*P*>0.100).

**Figure 2:**
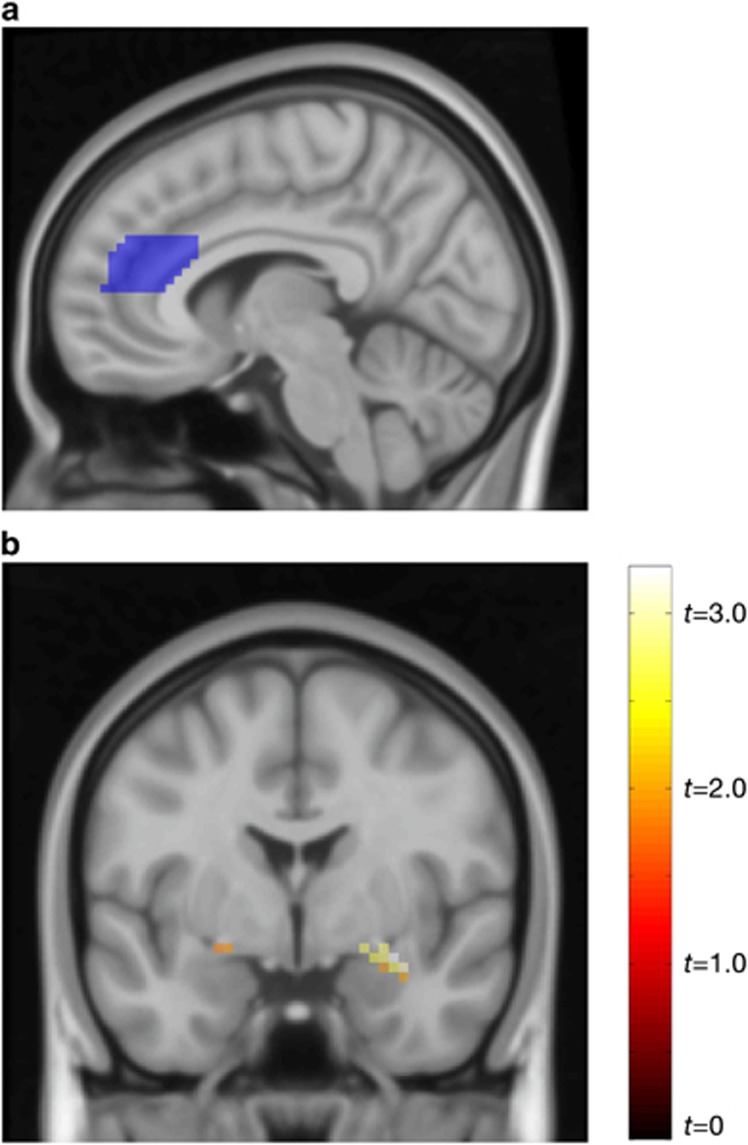
Statistical parametric map depicting less coupling between the dorsal anterior cingulate cortex (seed region) and the amygdala during self-directed criticism in responders as compared with nonresponders. (**a**) Sagittal view demonstrating the dorsal anterior cingulate cortex (dACC) mask used as the seed region in the psychophysiological interaction (PPI) analysis. (**b**) Coronal view showing amygdala task-dependent coupling with the dACC.

SVM analyses taking ACC subdivisions into account showed that the dACC alone (balanced accuracy 86.7%, confidence interval 95% 67.9–95.5, *P=*0.001, area under the receiver-operating characteristic curve (AUC)=0.97) and dACC together with the amygdala (balanced accuracy 91.7%, confidence interval 95% 73.2–97.6, *P=*0.001, AUC=0.89), but not the vACC, predicted 1-year treatment response. Furthermore, psychophysiological interaction analyses showed that the dACC, but not the vACC, was significantly (family-wise error, FWE corrected) less coupled with the right amygdala during self-referential criticism at pretreatment (*Z*=2.91, *P*^FWE^=0.036, 729 mm^3^, *x*,*y*,*z* [30,−1,−14], see also Figure 2) in long-term responders relative to nonresponders, with a similar tendency for dACC–left amygdala coupling (*Z*=1.81, *P*^uncorrected^=0.035, 54 mm^3^, *x*,*y*,*z* [−21,−1,−11]). Also, pretreatment voxel-wise univariate analyses suggested that the dACC was less reactive to self-referential criticism in responders as compared with nonresponders, although this was significant only when participants on concurrent medication were excluded from the analysis (*Z*=3.48, *P*^FWE^=0.030, 4320 mm^3^, *x*,*y*,*z* [12,32,28]).

### Supplementary analyses

Consistent with the main results, supplementary SVM analyses using the intention-to-treat approach revealed accurate prediction of 1-year iCBT responder status based on information from the ACC, and this region also remained a significant predictor when participants on concurrent psychotropic medication were excluded (Supplementary Material). Neither whole-brain nor ROI analyses using brain regions implicated in previous univariate SAD-fMRI prediction studies of CBT outcome^9, 10^ were predictive of long-term response, and diagnostic status, according to the structured clinical interview for DSM-IV, could not be predicted. Descriptive characteristics in Table 1 and scanner movement parameters did not differ between responders and nonresponders at pretreatment. Also, multiple logistic regressions using clinical and demographic variables did not reveal significant associations with long-term response to iCBT (see Supplementary Material).

## Discussion

By use of fMRI and SVM classification, we demonstrate that initial multivariate patterns of BOLD response to self-referential criticism in the ACC accurately predicted long-term response to iCBT. Further analyses showed that particularly the dorsal part of the ACC, together with the amygdala, predicted 1-year responder status, and that the initial coupling between these regions, as measured by psychophysiological interactions, was significantly lower in responders relative to nonresponders.

Thus, in individuals with SAD, treatment response 1 year after iCBT was accurately predicted by initial multi-voxel patterns of BOLD response to self-referential criticism in the ACC (92% balanced accuracy, defined as the mean of sensitivity and specificity). Our results are consistent with the notion that ACC-dependent self-referential processing is relevant for treatment outcome.^45^ Our long-term ACC findings are also coherent with prior studies using mass-univariate approaches or SVM to predict short-term response to CBT for SAD,^10^ panic disorder^17^ and depression.^18, 19, 45^ The ACC is, however, a functionally heterogeneous region linked to diverse processes such as conflict monitoring,^46^ action-outcome evaluation,^47^ fear expression^48^ and emotion regulation.^43^ Future studies are needed to determine whether such processes are involved in the current experimental task.^27^ Meanwhile, refined analyses in this study revealed that the dACC, in contrast to the vACC, was highly predictive, both by itself and together with the amygdala (92% balanced accuracy). Also, voxel-wise univariate analyses suggested lower initial dACC–amygdala coupling, and arguably also lower initial dACC reactivity to self-referential criticism, in responders relative to nonresponders. Both the dACC and the amygdala have been associated with fear expression.^43, 48, 49^ For instance, Vogt *et al.*^50^ examined the whole cingulate cortex and showed that 60% of the fear-induced activity was located in the dorsal ACC subregion, suggesting that multivariate brain activation patterns particularly in fear-expressing regions may influence sustained success of CBT.

Prior studies using clinical predictors of treatment response, such as symptom severity, have reported mixed results in SAD.^51^ The supplementary analyses in the present study showed that clinical and demographic variables, in contrast to SVM-fMRI, failed to predict iCBT long-term outcome, consistent with the notion that neuroimaging biomarkers add substantially to the predictive value of conventional factors for clinical treatment response.^9^ This was also noted in an fMRI random forest study on CBT outcome predictors in generalized anxiety disorder and panic disorder.^52^ The clinical utility of the multivariate SVM method may be substantial as it can predict accurately at the individual level. An important feature is that this method provides approximately unbiased estimates of generalizability to new cases. This was achieved by training the models on one sample and testing them on another independent sample, here realized using leave-one-out cross-validation. This represents a first step toward personalized treatment where therapeutic choice is guided by neuroimaging biomarkers. An important next step toward this objective is to validate the SVM models derived here using larger data sets, preferably derived from different sites and/or scanners.

There are several limitations of this study. Because of the small sample size, the results should be interpreted with some caution even though our sample size is in the higher range in comparison with other neuroimaging treatment prediction studies in the anxiety disorders.^20^ Unfortunately, reliability assessment of the CGI-I was not performed although the long-term clinical outcome is comparable to previous large-scale randomized controlled trials of iCBT for SAD (including 1-year follow-ups).^5, 28^ In contrast to the CGI-I, we were not able to predict improvement on the LSAS-SR continuous outcome and prediction of SAD diagnostic status, using the structured clinical interview for DSM-IV, was also not significant, suggesting that treatment outcome measures should be carefully selected in SVM prediction studies. The dACC–amygdala activity may be predictive of change in global symptoms rather than specific social anxiety symptoms, although other reasons for the discrepancy should be noted, such as the use of categorical vs continuous measures, and SVM vs relevance vector regression machine-learning algorithms. Only 13 participants were treatment-naive at the first fMRI assessment, and possible drug × iCBT interactions cannot be ruled out entirely. However, the distribution of participants on concurrent psychotropic medication was not different between responders and nonresponders and, importantly, the accurate predictions from the ACC remained significant when participants on concurrent SSRI treatment were excluded from the main SVM analyses (see Supplementary Material). Also, the supplementary intention-to-treat approach yielded consistent significant predictions by the ACC. The generalizability of the present study, however, may be limited because the SVM classification algorithm has not been applied to a separate clinical sample and our results may also be specific for iCBT and/or the specific fMRI task. Thus, future SVM research should investigate the predictive power of different experimental tasks, including resting-state fMRI, on CBT delivered both via the Internet and face-to-face.

In conclusion, we demonstrate that the initial dACC–amygdala BOLD response pattern to self-referential criticism is predictive of long-term treatment response to iCBT, and that lower pretreatment coupling between these fear-expressing brain regions is associated with stable symptom improvement. In contrast, clinical or demographic variables were not associated with sustained treatment response, underscoring the importance of taking into account neuroimaging biomarkers in prediction studies. To our knowledge, this is the first study using the multivariate SVM–fMRI method to successfully predict long-term treatment response in an anxiety disorder. Although SVMs hold high promise, additional studies involving the integration of imaging and related data from more than one site will allow us to further evaluate the clinical utility of this approach.

## Figures and Tables

**Table 1:** Demographic and clinical characteristics of study participants

	*All participants (*n=*26)*	*Responders at 1 year (*n=*12)*	*Nonresponders at 1 year (*n=*11)*	*Responders vs nonresponders at 1 year (*n=*12/11)*
Age (years), mean (s.d.)	32.3 (9.6)	35.5 (8.5)	31.5 (10.3)	*t*(21)=1.01, *P=*0.837
Range (years)	19–57	21–47	20–57	
Gender, female (%)	22 (85)	9 (75)	10 (91)	*χ*^2^(1)=1.01, *P=*0.315
Married or *de facto*, *n* (%)	15 (58)	7 (58)	7 (64)	*χ*^2^(1)=0.07, *P=*0.795
Educational level, *n* (%)				Fisher's exact *P=*0.386
Completed university	9 (35)	5 (42)	3 (27)	
Current university	10 (38)	5 (42)	3 (27)	
Lower grade^a^	7 (27)	2 (17)	5 (45)	
Psychotropic medication, *n* (%)	8 (31)	3 (25)	4 (36)	*χ*^2^(1)=0.35, *P=*0.554
Age of SAD onset (years), mean (s.d.)	15.9 (6.0)	16.3 (4.2)	16.6 (7.7)	*t*(21)=0.12, *P=*0.907
Pretreatment LSAS-SR, mean (s.d.)	76.3 (18.7)	74.1 (15.1)	77.6 (23.9)	*t*(21)=0.43, *P=*0.672
Pretreatment MADRS-S, mean (s.d.)	15.8 (6.6)	15.4 (8.1)	15.4 (5.8)	*t*(21)=−0.02, *P=*0.986

Abbreviations: LSAS-SR, Liebowitz Social Anxiety Scale—Self-report; MADRS-S, Montgomery Åsberg Depression Rating Scale—Self-report; SAD, social anxiety disorder.

a Including high school, vocational school and compulsory school.

**Table 2:** Predictions of clinical outcome at 1-year follow-up. The sensitivity, specificity and balanced classification accuracy (arithmetic mean of sensitivity and specificity) are presented as percentages

	*Balanced accuracy*	P *(balanced)*^a^	*Sensitivity*	*Specificity*	*AUC*
**ACC**	**91.7**	**0.001**	**83.3**	**100.0**	**0.91**
Amygdala	47.7	0.531	50.0	45.5	0.46
dlPFC	43.2	0.638	50.0	36.4	0.46
Hippocampus	51.9	0.412	58.3	45.5	0.37
Insula	43.6	0.592	41.7	45.5	0.45
vmPFC	39.0	0.694	41.7	36.4	0.29

Abbreviations: ACC, anterior cingulate cortex; AUC, area under the receiver-operating characteristic curve; dlPFC, dorsolateral prefrontal cortex; vmPFC, ventromedial prefrontal cortex.

a *P*-values are calculated from permutation testing with 1000 permutations. Significant balanced accuracies are in bold.
