# Supplementary material for: Predicting long-term outcome of Internet-delivered cognitive behavior therapy for social anxiety disorder using fMRI and support vector machine learning
Source: Transl Psychiatry. 2015 Mar 17;5(3):e530–. doi: 10.1038/tp.2015.22 (PMC4354352; doi:10.1038/tp.2015.22)
Supplement: Supplementary Information [file tp201522x1.doc]

Supplemental Material

Included elements: Figure S1, flow diagram showing included and excluded participants. Table S1 shows results from supplementary support vector machine (SVM) classifications.

### Supplementary Methods

### Treatment

The Internet-delivered Cognitive behavior therapy (CBT) included 9 separate modules, and each module was presented to the social anxiety disorder (SAD) participant each week during the treatment period. Also, included in each module, we implemented a multiple-choice quiz on which the patient needed to get 95% correct answers to proceed. After the participant handed in the homework and completed the quiz, the therapist gave written feedback on the homework to reinforce and modify the patients’ work, and then the next self-help module was introduced.

The first module contained an introduction to CBT and facts about SAD. Module 2-4 described the cognitive model for SAD and included tasks on cognitive restructuring. Modules 5-7 were mainly focusing on exposure exercises, and attention training. The last 2 modules provided information on social skills training, and relapse prevention. The treatment is published as a self-help book, and available in Swedish language.[^1^](#_ENREF_1)

The Attention Bias Modification trials always began with a fixation cross (“+”) presented in the center of the screen for 500 milliseconds. Subsequently, two facial expressions of the same person were displayed for 500 milliseconds. In 128 trials, these were neutral-disgust, and in the remaining 32 trials (*N*=160), neutral-neutral expressions were shown. The faces were presented at the top or at the bottom of the screen. Thereafter, a probe appeared in the location of the neutral face. Patients were instructed to indicate whether the probe was the letter E or F by pressing the corresponding arrow on the keyboard using their right hand. The probe remained on the screen until a response was given, after which the next trial began. For more details see Carlbring *et al.*[^2^](#_ENREF_2)

### Data Analysis

Follow-up analyses to the significant results from the ACC included intention-to-treat analysis, coding all dropouts as nonresponders, as well as removing all participants on concurrent medication. In order to replicate previous SAD-fMRI prediction studies to CBT using a multivariate method (i.e., SVM) we created spherical masks in the WakeForest University Pickatlas[^5^](#_ENREF_5) with 10mm radii centered at the following MNI-coordinates: superior temporal/angular gyrus xyz[−58,−66,28], medial orbitofrontal gyrus xyz[8,56,−8], middle temporal gyrus xyz[66,−10,−24],[^4^](#_ENREF_4) dorsal occipitotemporal cortex xyz[26,−87,20], and ventral occipitotemporal cortex xyz[42,−75,−12].[^3^](#_ENREF_3) SVM predictions of treatment outcome were conducted using blood oxygenation level-dependent (BOLD) response to self-referential processing separately for these masks, as well as for the whole brain. In addition, significant analyses from the main SVM-predictions on response rate, as determined by the Clinical Global Impression-Improvement scale (CGI-I), guided the selection of subsequent predictions on diagnostic status according to the SCID interview.[^6^](#_ENREF_6)

Also, as predictors of 1-year clinical response, we entered participants’ initial clinical data into logistic regression analyses, i.e. social anxiety severity (i.e., Liebowitz Social Anxiety Scale – Self-report version, depressive symptoms (i.e., [Montgomery Åsberg Depression Rating Scale – Self-report version, MADRS-^S;9^](#_ENREF_9) perceived quality of life (i.e., [Quality of Life Inventory, QOL^I;10^](#_ENREF_10) and clinical anxiety (i.e., [Beck Anxiety Inventory, BA^I;11^](#_ENREF_11) Similarly, in a separate regression model, we entered demographic variables as predictors, i.e., age, educational level, marital status, and sex.

### Supplementary Results

### Support Vector Machine Classifications

SVM classifications using the intention-to-treat (ITT) approach showed that the ACC accurately classified responder status at 1-year follow-up (88.7% balanced accuracy, *P*=0.001, area under the receiver operating characteristic curve, AUC=0.91). The ACC remained a significant predictor of long-term outcome when all participants on concurrent psychotropic medication (i.e., SSRIs) were excluded from the ITT analysis (ACC: 88.9% balanced accuracy, *P*=0.002, AUC=0.90) as well as the main (as-treated) analysis (ACC: 74.6% balanced accuracy, *P*=0.050, AUC=0.87).

SVMs based on the 2 previous SAD-fMRI prediction studies on CBT, and the whole-brain, are reported in Table S1.

In total, 43% (10/23) of all participants, and 67% (8/12) of the CGI-I responders did not fulfill the diagnostic criteria for SAD at 1-year follow-up. Initial BOLD response (i.e., in ACC, dorsal ACC, or dorsal ACC + amygdala) did not significantly predict those who did or did not fulfill the criterion for SAD, e.g., by the use of the ITT approach, the dorsal ACC (dACC) was insignificant in classifying 1-year diagnostic status (dACC: balanced accuracy: 64.4%, AUC=0.67), all *P´s*>0.102.

### Clinical and Demographic Characteristics

Neither clinical, nor movement parameters were significantly different between responders and nonresponders (all *P*´s>0.325). We found no significant baseline differences (all *P’s*>0.849) on clinical variables between compliant participants and those who withdrew (*N*=3) from the study.

Logistic regression analyses showed that initial social anxiety severity, depressive symptoms, perceived quality of life, and general anxiety were not predictive of 1-year treatment response (Adjusted R^2^=−0.21, *F*(4,18)=0.06, *P*=0.991), neither were demographic variables, i.e., age, gender, marital status, and educational level, (Adjusted R^2^=0.05, *F*(4,18)=1.22, *P*=0.317).

**Table S1.** Predictions of clinical outcome at long-term follow-up. The sensitivity, specificity and balanced classification accuracy (arithmetic mean of sensitivity and specificity) are presented as percentages

|  | Balanced Accuracy | *P*(Balanced)^a^ | Sensitivity | Specificity | AUC^b^ |
| --- | --- | --- | --- | --- | --- |
|  | | |  | | |
| dOCC | 30.3 | 0.920 | 33.3 | 27.3 | 0.32 |
| mOFC | 51.5 | 0.390 | 66.7 | 36.4 | 0.39 |
| MTG | 39.0 | 0.700 | 41.7 | 36.4 | 0.31 |
| STG/Angular gyrus | 69.3 | 0.070 | 75.0 | 63.6 | 0.64 |
| vOCC | 34.1 | 0.860 | 50.0 | 18.2 | 0.28 |
| Whole-brain | 43.2 | 0.596 | 50.0 | 36.4 | 0.45 |

^a^ *P*-values are calculated from permutation testing with 1000 permutations

^b^ AUC, Area under the receiver operating characteristic curve

Abbreviations: dOCC, Dorsal occipitotemporal gyrus; MTG, Middle temporal gyrus; mOFC, Medial orbitofrontal cortex; STG, Superior temporal gyrus; vOCC, Ventro occipitotemporal cortex

**
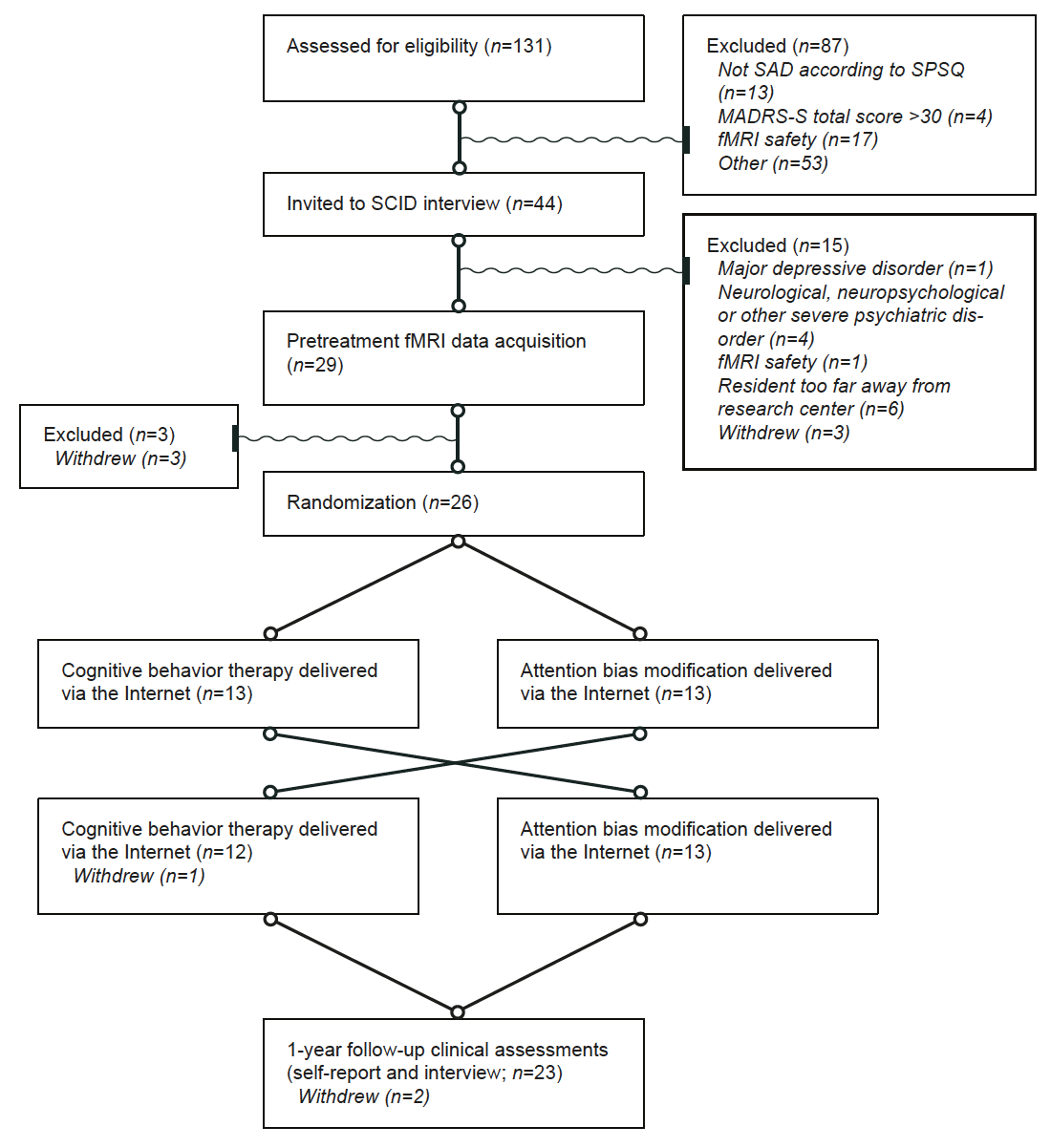
**

**Figure S1.** Flow diagram showing included and excluded participants

Abbreviations: fMRI, Functional magnetic resonance imaging; MADRS-S, Montgomery Asberg Depression Rating Scale – Self-report version; SAD, Social anxiety disorder; SPSQ, Social Phobia Screening Questionnaire

**Supplementary References**

1. Furmark T, Holmström A, Sparthan E, Carlbring P, Andersson G. *Social fobi - Effektiv hjälp med kognitiv beteendeterapi [Social Anxiety - Effective treatment using cognitive behavioural therapy]*, 2nd edn. Liber: Stockholm, 2006.

2. Carlbring P, Apelstrand M, Sehlin H, Amir N, Rousseau A, Hofmann SG*, et al*. Internet-delivered attention bias modification training in individuals with social anxiety disorder: A double blind randomized controlled trial. *BMC Psychiatry* 2012; **12:** 66.

3. Doehrmann O, Ghosh S, Polli F, Reynolds G, Horn F, Keshavan A*, et al*. Predicting treatment response in social anxiety disorder from functional magnetic resonance imaging. *JAMA Psychiatry* 2012; **70:** 87-97.

4. Klumpp H, Fitzgerald DA, Phan KL. Neural predictors and mechanisms of cognitive behavioral therapy on threat processing in social anxiety disorder. *Prog Neuropsychopharmacol Biol Psychiatry* 2013; **45:** 83-91.

5. Maldjian JA, Laurienti PJ, Kraft RA, Burdette JH. An automated method for neuroanatomic and cytoarchitectonic atlas-based interrogation of fMRI data sets. *NeuroImage* 2003; **19:** 1233-1239.

6. First M, Gibbon M, Spitzer R, Williams J. *Structured clinical interview for DSM–IV axis I disorders (SCID–I)*. American Psychiatric Press: Washington, 1997.

7. Baker SLS, Heinrichs NN, Kim H-JH, Hofmann SGS. The liebowitz social anxiety scale as a self-report instrument: A preliminary psychometric analysis. *Behav Res Ther* 2002; **40:** 701-715.

8. Liebowitz MR. Social phobia. *Mod Probl Pharmacopsychiatry* 1987; **22:** 141-173.

9. Svanborg P, Åsberg M. A new self-rating scale for depression and anxiety states based on the comprehensive psychopathological rating scale. *Acta Psychiatr Scand* 1994; **89:** 21-28.

10. Frisch MB, Cornell J, Villanueva M, Retzlaff PJ. Clinical validation of the quality of life inventory: A measure of life satisfaction for use in treatment planning and outcome assessment. *Psychol Assessment* 1992; **4:** 92-101.

11. Beck AT, Epstein N, Brown G, Steer RA. An inventory for measuring clinical anxiety: Psychometric properties. *J Consult Clin Psychol* 1988; **56:** 893-897.
